# Lynch syndrome-associated endometrial carcinoma with *MLH1* germline mutation and *MLH1* promoter hypermethylation: a case report and literature review

**DOI:** 10.1186/s12885-018-4489-0

**Published:** 2018-05-21

**Authors:** Takanori Yokoyama, Kazuhiro Takehara, Nao Sugimoto, Keika Kaneko, Etsuko Fujimoto, Mika Okazawa-Sakai, Shinichi Okame, Yuko Shiroyama, Takashi Yokoyama, Norihiro Teramoto, Shozo Ohsumi, Shinya Saito, Kazuho Imai, Kokichi Sugano

**Affiliations:** 1 Department of Gynecology, National Hospital Organization Shikoku Cancer Center, Minamiumemoto-cho, Matsuyama-city, Ehime Prefecture 791-0280 Japan; 2 Counseling Room of Familial Neoplasm, National Hospital Organization Shikoku Cancer center, Minamiumemoto-cho, Matsuyama-city, Ehime Prefecture 791-0280 Japan; 3 Department of Pathology, National Hospital Organization Shikoku Cancer center, Minamiumemoto-cho, Matsuyama-city, Ehime Prefecture 791-0280 Japan; 4 Oncogene Research Unit/Cancer Prevention Unit, Tochigi Cancer Center Research Institute, Tochigi, Japan

**Keywords:** Lynch syndrome, Endometrial cancer, *MLH1* promoter hypermethylation, *MLH1* germline mutation, Screening

## Abstract

**Background:**

Lynch syndrome is an autosomal dominant inherited disease caused by germline mutations in mismatch repair genes. Analysis for microsatellite instability (MSI) and immunohistochemistry (IHC) of protein expressions of disease-associated genes is used to screen for Lynch syndrome in endometrial cancer patients. When losses of both MLH1 and PMS2 proteins are observed by IHC, *MLH1* promoter methylation analysis is conducted to distinguish Lynch syndrome-associated endometrial cancer from sporadic cancer.

**Case presentation:**

Here we report a woman who developed endometrial cancer at the age of 49 years. She had a family history of colorectal cancer (first-degree relative aged 52 years) and stomach cancer (second-degree relative with the age of onset unknown). No other family history was present, and she failed to meet the Amsterdam II criteria for the diagnosis of Lynch syndrome. Losses of MLH1 and PMS2, but not MSH2 and MSH6, proteins were observed by IHC in endometrial cancer tissues. Because *MLH1* promoter hypermethylation was detected in endometrial cancer tissue samples, the epigenetic silencing of *MLH1* was suspected as the cause of the protein loss. However, because of the early onset of endometrial cancer and the positive family history, a diagnosis of Lynch syndrome was also suspected. Therefore, we provided her with genetic counseling. After obtaining her consent, *MLH1* promoter methylation testing and genetic testing of peripheral blood were performed. *MLH1* promoter methylation was not observed in peripheral blood. However, genetic testing revealed a large deletion of exon 5 in *MLH1*; thus, we diagnosed the presence of Lynch syndrome.

**Conclusions:**

Both *MLH1* germline mutation and *MLH1* promoter hypermethylation may be observed in endometrial cancer. Therefore, even if *MLH1* promoter hypermethylation is detected, a diagnosis of Lynch syndrome cannot be excluded.

## Background

Lynch syndrome is an autosomal dominant inherited disease caused by germline mutations in mismatch repair genes. *MLH1, MSH2, MSH6, PMS2* mutation in this syndrome account for approximately 37, 41, 13, 9%, respectively [1]. It is important to establish a diagnosis for this syndrome because of the associated elevated lifetime risk of developing cancers such as colorectal and endometrial cancers [2]. Amsterdam II [3] and Bethesda [4] criteria have been widely used to screen for Lynch syndrome. However, the sensitivity of both criteria based on family history and clinical background is low [5–7]. Therefore, they have been considered to be insufficient as independent screening tools. To increase the accuracy of screening for Lynch syndrome, microsatellite instability (MSI) and immunohistochemistry (IHC) are used in cases with colorectal cancer, and when losses of MLH1 and PMS2 proteins are detected using IHC, universal screening, including *BRAF* testing and analysis for *MLH1* promoter methylation, is recommended [5, 6]. Similarly, in endometrial cancer, MSI and IHC are useful in screening for Lynch syndrome [7–10], and the mutation site can be estimated using IHC. When losses of MLH1 and PMS2 proteins are observed, both *MLH1* germline mutations and epigenetic silencing are conceivable. *MLH1* promoter methylation testing is recognized to be useful for distinguishing between the two in the population based screening, because *MLH1* promoter hypermethylation is far more responsible for losses of MLH1 and PMS2 protein using IHC than *MLH1* germline mutation [7]. However, some reports showed cases with germline mutations among Lynch syndrome patients with colorectal cancer in which although MLH1 and PMS2 proteins were lost by IHC in cancer tissues, *MLH1* promoter hypermethylation was observed [11–13]. These reports suggested that it is inappropriate to exclude Lynch syndrome based on a result of *MLH1* promoter hypermethylation. Similar assumption is made for Lynch syndrome associated with endometrial cancer. However, to the best of our knowledge, we have been unsuccessful in finding any previous study similar to the present case report.

In this study, we performed genetic analysis of peripheral blood from this endometrial cancer patient with MLH1 and PMS2 protein losses using IHC despite *MLH1* promoter hypermethylation because Lynch syndrome was suspected based on family history and clinical factors. As a result, we confirmed the presence of *MLH1* germline mutation, and hence, diagnosed this patient with Lynch syndrome.

## Case presentation

Here we report a woman who developed endometrial cancer at the age of 49 years, with no previous history of cancer. She had a family history of a first-degree relative who developed colorectal cancer at the age of 52 years and a second-degree relative who developed stomach cancer at an unknown age of onset. No other relevant family history was revealed, and she did not meet the Amsterdam II criteria for the diagnosis of Lynch syndrome. She visited our hospital with a chief complaint of atypical genital bleeding. A diagnosis of endometrial cancer was made, and we performed hysterectomy and salpingo-oophorectomy and partial omentectomy. Based on the histopathological examination of samples obtained at surgery, a grade 2 endometrioid adenocarcinoma in the endometrium was diagnosed. Similarly, the pathological stage of the disease determined at surgery enabled an International Federation of Gynecology and Obstetrics (2008) stage IA disease to be established.

**Fig. 1:**
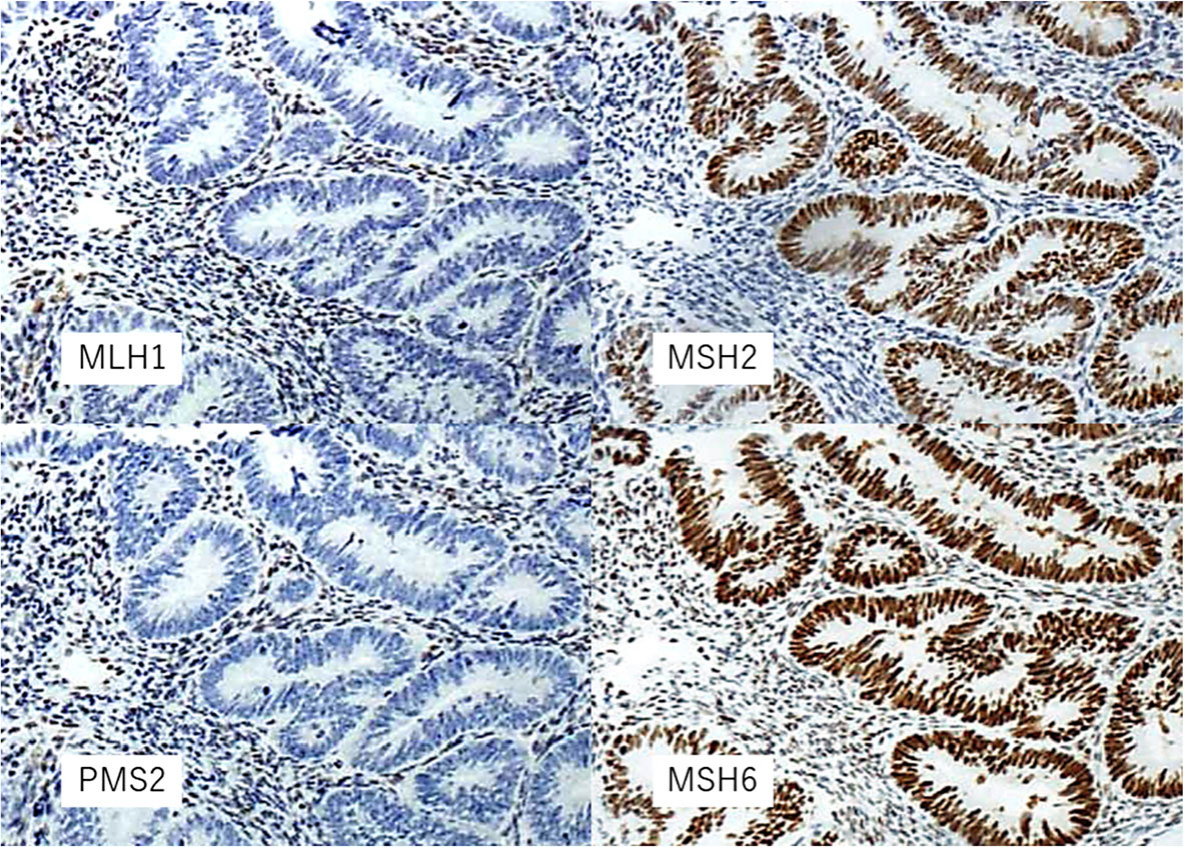
Immunohistochemistry. Losses of MLH1 and PMS2 proteins were detected in the tumor cells. Expressions of MSH2 and MSH6 proteins were detected in the tumor cells. Expressions of MLH1, PMS2, MSH2, MSH6 proteins were detected in the lymphocytes

As a result of our previous IHC analysis to screen for endometrial cancer [10] that revealed losses of MLH1 and PMS2, but not MSH2 and MSH6, proteins in endometrial cancer tissues obtained from this patient (Fig. 1), *MLH1* mutation was suspected. We confirmed *MLH1* promoter hypermethylation in endometrial cancer tissue samples and suspected epigenetic silencing of *MLH1*. However, because of the early age of onset of endometrial cancer and presence of family history of Lynch-related malignancies, we suspected her of showing Lynch syndrome; therefore, we offered her genetic counseling. After obtaining consent, *MLH1* promoter methylation and genetic testing of peripheral blood were performed. *MLH1* promoter methylation was not observed in peripheral blood. However, genetic testing revealed a large deletion in exon 5 of *MLH1*; therefore, we diagnosed this patient with Lynch syndrome.

## Discussions and conclusions

This report demonstrated that in endometrial cancer, Lynch syndrome cannot be excluded even if *MLH1* promoter hypermethylation is confirmed in cases with MLH1 and PMS2 protein loss detected using IHC.

Buchanan et al. proposed a strategy for screening for Lynch syndrome in endometrial cancer patients using MSI, IHC, and *MLH1* promoter methylation. They recommended performing IHC on tumor tissue samples from patients who develop endometrial cancer at or before 60 years of age [7]. *MLH1* germline mutation, epimutation [14], or epigenetic silencing can be considered when MLH1 and PMS2 protein losses are detected. Buchanan et al. reported that mismatch repair IHC tumor testing had high sensitivity and poor positive predictive value [7]. Therefore, it is necessary to conduct further examinations because of the poor positive predictive value. *BRAF* testing in colon cancer is useful to rule out Lynch syndrome [6]. However, the usefulness of *BRAF* testing to detect endometrial cancer is unclear because *BRAF* mutation is infrequently found [15]. *MLH1* promoter methylation testing is useful for distinguishing between *MLH1* germline mutation and epigenetic silencing. MLH1 promoter hypermethylation is far more responsible for losses of MLH1 and PMS2 protein using IHC than MLH1 germline mutation in the population based screening. Thus, cases with *MLH1* promoter hypermethylation in cancer tissue are generally classified as sporadic endometrial cancer with abnormal somatic mismatch repair gene [7].

However, recently some reports showed cases with germline mutations among colorectal cancer cases in which although MLH1 and PMS2 proteins were lost by IHC, *MLH1* promoter hypermethylation was observed in cancer tissues [11–13] (Table 1). These reports suggested that it is inappropriate to exclude Lynch syndrome based on a result of *MLH1* promoter hypermethylation. Hagen et al. reported that they confirmed *MSH2* germline mutation and somatic *MLH1* promoter hypermethylation in a 71-year-old female with confirmed MLH1, MSH2, MSH6, and PMS2 protein losses in the colon cancer tissues using IHC [11]. Raymond et al. also reported confirmed *MSH6* germline mutation and somatic *MLH1* promoter hypermethylation in a 75-year-old female with losses of MLH1, MSH6, and MSH2 proteins in the colon cancer tissue samples using IHC and concluded that *MLH1* promoter hypermethylation does not exclude the diagnosis of Lynch syndrome [12]. Rahner et al. examined *MLH1* promoter methylation from 60 carriers of *MLH1* germline mutation, 38 carriers of *MSH2* germline mutation, and 25 individuals without germline mutation. *MLH1* promoter methylation was observed in one carrier each of *MLH1* and *MSH2* germline mutations. Therefore, they concluded that *MLH1* promoter hypermethylation could not exclude the diagnosis of Lynch syndrome [13]. In the National Comprehensive Cancer Network guidelines, when losses of MLH1 and PMS2 proteins are detected using IHC in the presence of somatic *BRAF* mutations and *MLH1* promoter hypermethylation, it is recommended to consider genetic testing in cases of early onset and positive family history of Lynch-associated malignancies [5]. In the present case, sporadic endometrial cancer was suspected based on the results of IHC and *MLH1* promoter methylation testing of endometrial cancer tissues. *MLH1* promoter methylation was not observed in the peripheral blood; however, a large deletion in exon 5 of this gene was observed. Therefore, we diagnosed the patient with Lynch syndrome. With respect to endometrial cancer, the coexistence of *MLH1* germline mutation and *MLH1* promoter hypermethylation in the same patient was absent in the range of our literature search. However, similar to colorectal cancer, when screening for Lynch syndrome using endometrial cancer tissue, this is a pitfall that should not be overlooked.

**Table 1:** Co-existed MLH1 hypermethylation and mismatch repair genes germline mutations

		IHC							
	Tumor	MLH1	MSH2	MSH6	PMS2	MSI	BRAF V600E mutation	MLH1 promoter methylation	Germline mutation
Hagen et al. [11]	CRC	–	–	–	–	N/A	Wild-type	+	*MSH2* mutation
Raymond et al. [12]	CRC	–	+	–	–	MSI-H	Wild-type	+	*MSH6* mutation
Rahner et al. [13]	CRC	–	+	+	–	MSI-H	N/A	+	*MLH1* mutation
Yokoyama et al.	EC	–	+	+	–	N/A	N/A	+	*MLH1* mutation

*CRC* colorecral cancer, *EC* uterine endometrial cancer, *N/A* not available

In summary, we have demonstrated that in endometrial cancer, similar to colorectal cancer, both *MLH1* germline mutation and somatic *MLH1* promoter hypermethylation may be observed in the same patient and that Lynch syndrome cannot be excluded even if *MLH1* promoter hypermethylation is observed. From a cost perspective, it is considered prohibitive to conduct genetic testing for all cases that present with *MLH1* promoter hypermethylation. However, it has been suggested that genetic testing should be considered in endometrial cancer patients with *MLH1* promoter hypermethylation at least if clinical and family histories are indicative of Lynch syndrome.

## Abbreviations

IHC: Immunohistochemistry
MSI: Microsatellite instability
N/A: Not available
